# Foliar cytokinins or brassinosteroids applications influence the rice plant acclimatization to combined heat stress

**DOI:** 10.3389/fpls.2022.983276

**Published:** 2022-12-22

**Authors:** Alvaro Daniel Pantoja-Benavides, Gabriel Garces-Varon, Hermann Restrepo-Díaz

**Affiliations:** ^1^ Universidad Nacional de Colombia, Sede Bogotá, Facultad de Ciencias Agrarias, Departamento de Agronomía, Bogotá, Colombia; ^2^ Federación Nacional de Arroceros, Seccional Saldaña, Saldaña, Colombia

**Keywords:** Fv/Fm ratio, high daytime and nighttime temperature, lipid peroxidation, plant acclimatization, phenological stages, stomatal conductance

## Abstract

The effect of different foliar sprays numbers of cytokinins – (CK) and brassinosteroids – (BR) on the physiological, biochemical, and panicle parameters of rice plants subjected to combined heat stress (high day/night temperatures) were studied in three different experiments. The treatments established for the first (E1) and second (E2) experiments were the following: i) absolute control, ii) stress control, iii) heat stress + one foliar spray of CK, iv) heat stress + two foliar sprays of CK, v) heat stress + three foliar sprays of CK, vi) heat stress + one foliar spray of BR, vii) heat stress + two foliar sprays of BR, or viii) heat stress + three foliar sprays of BR. For the third experiment (E3), the treatments were the following: i) absolute control, ii) stress control, iii) heat stress + three foliar applications of CK, iv) heat stress + three foliar applications of BR. Rice-stressed plants and sprayed with three foliar sprays of CK or BR had a better stomatal conductance in E1 and E2 compared to their heat-stressed control. The relative tolerance index suggests that three CK or BR applications helped to mitigate the combined heat stress in both experiments. The foliar CK or BR applications at the flowering and grain-filling stages in rice-stressed plants increased Fv/Fm ratio and panicle characteristics (number of filled spikelets and the percentage of panicle blanking in E3). In conclusion, foliar applications of BR or CK can be considered an agronomic strategy to help improve the negative effect of combined heat stress conditions on the physiological behavior of rice plants during different phenological stages.

## 1 Introduction

Rice is a key food crop that is produced in many countries around the world and feeds a large part of the world’s population (IRRI, 2006; Kaur and Pati, 2017). This crop is important for global food security since it is consumed by most of the world’s poor people (Samal and Babu, 2018; Kandil et al., 2022). Rice demand is expected to increase with population growth by 2050 and reach values of 607 million tons based on current consumption (Samal and Babu, 2018; Gérardeaux et al., 2021). Local rice cultivation has a great economic value due to its high consumption and large extensions throughout the Colombian territory (Garcés, 2020). In Colombia, rice occupied 617,934 ha with a national production volume of 2,937,840 t and an average yield of 5.02 t ha^-1^ in 2020 (Fedearroz, 2021).

Climate change has generated an increase in temperature, exposing most crops (including rice) to conditions of heat stress during their sensitive phenological stages, which this stressor is a serious challenge that needs to be addressed (Sharma et al., 2017; Hassan et al., 2022). Higher temperatures have caused a 6-7% decrease in the production of rice crops in recent years (Lesk et al., 2016). Additionally, climatic variability phenomena (such as El Niño Southern Oscillation (ENSO) phenomenon) can generate episodes of heat stress in some regions, affecting crops (Iizumi et al., 2014; Amanullah et al., 2017).

In Colombia, rice-producing areas are expected to suffer increases in temperatures of between 2°C and 2.5°C by 2050, which will reduce rice yields and have an impact on the flows of agricultural products to markets and supply chains (Castro-Llanos et al., 2019). High temperatures, in general, are detrimental to most physiological processes such as stomatal opening, photosynthesis, growth, and grain yield (Ahammed et al., 2015). Studies on stress caused by high temperatures have mainly focused on the reproductive stage due to its high sensitivity and the relationship between rice flag leaf behavior and grain yield (Das et al., 2014; Hatfield and Prueger, 2015; Wu et al., 2016). Therefore, research on how the functioning of the flag leaf is affected by stress, mainly at the reproductive stage of rice, has been carried out (Restrepo-Diaz and Garces-Varon, 2013). Karwa et al. (2020) and Zhang et al. (2016) observed that the gas exchange properties and chlorophyll fluorescence parameters of the flag leaf (before entering the reproductive stage) of rice cultivars decreased when plants were subjected to heat stress conditions.

The use of growth regulators, or biostimulants, has been evaluated as an agronomic management strategy to mitigate the negative effects caused by environmental stress in recent years (Ahammed et al., 2015; Quintero-Calderón et al., 2021; Akhtar et al., 2022). Fahad et al. (2016) observed that the use of growth regulators (including brassinosteroids) helped rice plants to acclimatize to the heat stress condition, improving stomatal conductance, photosynthesis, and transpiration. Kosakivska et al. (2022) also found that exogenous applications of cytokinins favored stomatal conductance, photosynthesis, and different photosynthetic pigments in different cereals subjected to heat stress. In Colombia, previous studies have shown that foliar applications of brassinosteroids or biostimulants (with cytokinins in their formulation) mitigated the effects of heat stress in rice plants since these substances improved the gas exchange properties of leaves and the contents of chlorophyll and proline (Quintero-Calderon et al., 2021).

Cytokinins (CK) are growth regulators that generate plant responses to the adverse effects caused by abiotic and biotic stresses. Because of this, their use can be considered a tool to mitigate the damage caused to physiological and biochemical mechanisms (Veselov et al., 2017). The number of exogenous applications of CK has been reported as a factor to consider in the reduction of damage caused by high temperatures (Veselov et al., 2017). For example, three foliar sprays of cytokinins to creeping bentgrass (*Agrostis stolonifera*) plants at different development stages have been observed to delay leaf senescence and improve tolerance to heat stress by increasing antioxidant activities and decreasing lipid peroxidation (Veerasamy et al., 2007). Wu et al. (2016) also found that plants of a rice variety susceptible to heat stress improved their physiological activity under a stress condition with two applications of 6-benzylaminopurine. Additionally, a single application of CK (zeatin) helped the antioxidant activity and synthesis of different enzymes in chickpea under heat stress, decreasing the damage of reactive oxygen species (ROS) and the production of malondialdehyde (MDA) (Kumar et al., 2020). Finally, exogenous cytokinin treatment usually improves heat stress resistance because exogenous applications can upregulate heat shock proteins; indicating that cytokinin is capable of priming heat stress defense (Mandal et al., 2022).

Brassinosteroids (BR) are growth regulators that have also been used to mitigate the effects of different abiotic stresses, including high-temperature stress (Kothari and Lachowiec, 2021). Thussagunpanit et al. (2015) showed that when BR were applied, they generated tolerance to heat stress by protecting the photosynthetic machinery (favoring the photosynthetic rate and stomatal conductance and reducing photoinhibition) in rice plants during the reproductive stage. An exogenous application of BR also accelerated the acclimatization process to high temperatures and generated thermotolerance since it increased the survival rate of wheat seedlings (Hairat and Khurana, 2015). In the same research, Hairat and Khurana (2015) also report an improvement in the photosynthetic rate and a decrease in membrane damage in plants exposed to heat stress and treated with BR compared to untreated plants. A study by Sonjaroon et al. (2018) showed that rice plants treated with an application of 7,8-dihydro-8α-20-hydroxyecdysone (αDHECD, 0.0001 µM) (synthetic brassinosteroid) increased their photosynthetic parameters under heat stress compared to untreated and stressed plants. Br-induced heat tolerance could be associated with the upregulation of transcript levels of *RBOH1*, *MPK1*, and *MPK2* genes, enhancing antioxidant activity in the plant (Ahammed et al., 2016).

Rice crops have recently suffered periods of high daytime and nighttime temperatures due to climate change and variability in different regions of the world (including Colombia) (Sharma et al., 2017; Garcés, 2020). The use of different types of biostimulants (including compounds with nutrients and growth regulators) has recently been evaluated as an agronomic strategy to mitigate heat stress in rice-growing areas at local and global levels (Sonjaroon et al., 2018; Calderón-Paez et al., 2021; Quintero-Calderón et al., 2021). On the other hand, the use of biochemical (malondialdehyde and proline synthesis) and physiological (leaf canopy temperature, stomatal conductance, chlorophyll fluorescence parameters, and relative chlorophyll and water contents) variables has been a tool to assess the efficiency of treatments to mitigate heat stress damage (Fahad et al., 2016; Sarsu et al., 2018). Foliar sprays of CK and BR can be a tool to help in the mitigation of heat stress. However, the effectiveness of this technique may be conditioned by parameters such as the concentration of the compound, the number of applications per crop cycle, or the stage of development of plants in which these compounds are applied (Kothari and Lachowiec, 2021). Therefore, the objective of this research was to evaluate the effect of foliar CK or BR sprays on physiological, biochemical, and panicle parameters of rice plants subjected to combined heat stress (high day/night temperatures) at different phenological stages.

## 2 Material and methods

### 2.1 Plant material and general growth conditions

Rice seeds of the genotype Fedearroz 2000 (F2000 - genotype released in the last decade of the 20th century due to its tolerance to the white leaf virus (WLV)) were used in the three experiments. This genotype is widely cultivated by Colombian farmers but has shown a high susceptibility to heat stress. The seeds were sown in 10 L trays (39.6 cm long x 28.8 cm wide x 16.8 cm high) containing sandy loam soil with 2% organic matter (soil from “Las Lagunas” research center). Five pregerminated seeds were sown in each tray. The trays for the first experiment (E1) were placed in a greenhouse at the Faculty of Agricultural Sciences of the Universidad Nacional de Colombia, Bogotá campus (43°50’56” N, 74°04’051” W), at an altitude 2,556 meters above sea level (masl), between December 2020 and February 2021. The environmental conditions in the greenhouse during this trial were as follows: average daytime temperature of 30°C, average nighttime temperature of 25°C, 60%-80% relative humidity, and a natural photoperiod of 12 h (photosynthetically active radiation of 1500 µmol (photons) m^-2^ s^-1^ at noon).

The second (E2) and third experiment (E3) were carried out under mesh house conditions at the “Las Lagunas” research center of the National Federation of Rice Growers (Fedearroz) located in Saldaña (Colombia) (3°54’46’’ N; 74°59’7’’ W) between December 2020 and April 2021 for E2 and between August and November 2021 for E3. The environmental conditions throughout the experiments were as follows: average daytime temperature of 33°C, average night temperature of 24°C, relative humidity of 77%, and natural photoperiod of 12 hours. These values were recorded by a weather station (Davis Vantage Pro-2 Plus, NSW, AUS) located in the experiment area. The plants were fertilized 20 days after seed emergence (DAE) in the three experiments, following the amounts of each element according to Sánchez-Reinoso et al. (2019) in experiments 2 and 3: 670 mg of N per plant, 110 mg of P per plant, 350 mg of K per plant, 68 mg of Ca per plant, 20 mg of Mg per plant, 20 mg of S per plant, 17 mg of Si per plant, 10 mg of B per plant, 17 mg of Cu per plant, and 44 mg of Zn per plant.

Rice plants were maintained under these general growth conditions until 47 DAE when they reached phenological stage V5. Different studies have shown that this phenological stage is the right moment to establish heat stress tests on rice plants (Sánchez-Reinoso et al., 2014; Alvarado-Sanabria et al., 2017). The combined heat stress treatments were established at 80 DAE (R2 - booting) in the second experiment. Regarding E3, the rice plants were also kept under mesh house conditions until 80 DAE when they reached phenological stage R2 (booting). At that time, a first group of plants was exposed to combined heat stress. Then, a second group of plants was also subjected to combined stress conditions at 100 days when they were at phenological stage R8 (grain filling). In experiments 2 and 3, the plants only had the main tiller with its corresponding flag leaf. Finally, the stress periods in the tests with adult plants were selected based on literature that states that rice plants are more sensitive to the effects of heat stress during these phenological stages (reproductive and maturation) (Restrepo-Diaz and Garces-Varon, 2013; Alvarado-Sanabria et al., 2017; Alvarado-Sanabria et al., 2021).

### 2.2 First experiment (E1): Evaluation of foliar sprays of cytokinins or brassinosteroids in rice seedlings

A preliminary experiment was carried out to evaluate the effect of the number of foliar sprays (1, 2, or 3 sprays) of two plant hormones (cytokinins vs. brassinosteroids) on the mitigation of combined heat stress in the seedling stage. The compounds to be applied were determined based on the results obtained in a previous experiment (Pantoja-Benavides et al., 2021). In this trial, rice plants of the genotype F2000 were initially divided into eight different treatment groups in which the conditions of heat stress and number of applications of CK and BR were combined. Finally, the established treatment groups were the following: i) absolute control (AC), ii) stress control (SC), iii) heat stress + one foliar spray of CK (CK1), iv) heat stress + two foliar sprays of CK (CK2), v) heat stress + three foliar sprays of CK (CK3), vi) heat stress + one foliar spray of BR (BR1), vii) heat stress + two foliar sprays of BR (BR2), or viii) heat stress + three foliar sprays of BR (BR3).

Foliar applications of CK used 97% pure trans-zeatin at a concentration of 1x10^-5^ M (AK Scientific, Union City, CA, USA). BR applications used an analog brassinosteroid ((25 R) – 3β. 5α – dihydroxy-spirostan-6-one) at a concentration of 5x10^-5^ M (Biomex DI-31, Minerales exclusivos S.A., Colombia). These doses were used in previous research where they showed a positive effect on the mitigation of combined heat stress in rice leaves (Pantoja-Benavides et al., 2021). Foliar sprays of these growth regulators were carried out using an atomizer with an application volume of 25 ml per plant, wetting both the upper and lower sides of leaves. Absolute control (AC) and stressed control (SC) plants were only sprayed with distilled water. All applications were performed at 09:00 h. Finally, the plants were arranged in a completely randomized design with five replicates per treatment and each replicate consisted of one plant. Each plant was used as a sampling unit for the corresponding readings of the variables determined at the end of the preliminary experiment. The experiment lasted 55 DAE.

### 2.3 Second experiment (E2): Effect of foliar sprays of cytokinins or brassinosteroids on the flag leaves of rice plants

Rice plants of the genotype Fedearroz 2000 were also used in this trial and arranged in eight different treatments as in the preliminary experiment (E1). This test also aimed to estimate the effect of the number of foliar applications of CK or BR on the physiological behavior of flag leaves subjected to combined heat stress. The different treatment groups were the following: i) absolute control (AC), ii) stress control (SC), iii) heat stress + one foliar spray of CK (CK1), iv) heat stress + two foliar sprays of CK (CK2), v) heat stress + three foliar sprays of CK (CK3), vi) heat stress + one foliar spray of BR (BR1), vii) heat stress + two foliar sprays of BR (BR2), or viii) heat stress + three foliar sprays of BR (BR3). The compounds and doses for the applications of CK or BR were the same as those used in E1. The different number of foliar sprays of the growth regulators and control treatments (distilled water) were performed at maximum tillering and beginning of floral primordium or booting so that the moments of application matched the agronomic practices of the crop. The foliar application of the treatments followed the same methodology indicated in E1. All treatments were also arranged in a completely randomized design with five replicates and each replicate consisted of one plant. Each plant was used as a sampling unit for the corresponding readings of the variables determined at the end of the experiment. The experiment lasted 88 DAE.

### 2.4 Third experiment (E3): Evaluation of foliar applications of cytokinins or brassinosteroids on rice plants at two reproductive stages

Rice plants of the genotype F2000 were subjected to combined heat stress at two different phenological stages (reproductive stage (R2) or maturation (R8)) to evaluate the effect of three foliar applications of CK or BR on the gas exchange properties of the flag leaf and characteristics of the panicle. Eight treatment groups were also established: i) absolute control at flowering or maturation (AC), ii) heat stress control at flowering or maturation (SC), iii) combined heat stress at flowering or maturation + three foliar applications of CK (CK3), and iv) combined heat stress at flowering or maturation + three foliar sprays of BR (BR3). The compounds and doses for the applications of CK and BR were the same used in E1. The different number of foliar applications of the growth regulators and control treatments (distilled water) were performed at maximum tillering and beginning of floral primordium or booting so that the moments of application matched the agronomic practices of the crop. The foliar application of the treatments followed the same methodology indicated in E1 and E2. All treatments were organized in a factorial arrangement where the main factor was the imposition of stress in the phenological stage (R2 or R8) and the secondary factor was the foliar sprays. Each treatment also had five replicates and each replicate consisted of one plant. Each plant was used as a sampling unit for the corresponding readings of the variables determined at the end of the experiment. The experiment lasted 120 DAE.

### 2.5 Combined heat stress treatment

Combined heat stress treatments were carried out on the following dates: 47 DAE for E1, 80 DAE for E2, and 80 or 100 DAE for E3. Rice seedlings (E1) were transferred from the greenhouse to growth chambers with a capacity of 294 L (MLR-351H, Sanyo, Illinois, USA) at 47 DAE. In E2, the rice plants from the mesh house were placed in growth chambers with a capacity of 698 L (KBW-720, Binder, Germany) at 80 DAE. The plants in E3 were also placed in a 698 L chamber at 80 DAE or 100 DAE, which corresponds to phenological stages R2 and R8, respectively. The plant material was placed in growth chambers to simulate heat stress conditions or continue with previous environmental conditions. For all experiments, the combined heat stress treatment was established by setting the chambers to the following day/night temperatures: a period of high daytime (40°C for 5 hours (from 11:00 to 16:00)) and night (30°C for 5 hours (from 19:00 to 24:00)) temperatures for eight consecutive days. Stress temperatures and exposure times were selected based on previous studies (Sánchez-Reinoso et al., 2014; Alvarado-Sanabria et al., 2017). On the other hand, a group of plants transferred to the growth chamber was kept at the same greenhouse temperature (30°C day/25°C night) for eight consecutive days (absolute controls).

### 2.6 Variables determined in experiments 1 and 2

For experiments 1 and 2, the following physiological and biochemical variables were determined:

#### 2.6.1 Stomatal conductance

Stomatal conductance (g_s_) was determined using a portable porometer (SC-1, METER Group Inc., USA) with a range from 0 to 1000 mmol m^–2^ s^−1^ and a sample chamber opening of 6.35 mm in both experiments. Readings were taken by placing the porometer sensor on a fully expanded mature leaf of the main tiller of the plant. g_s_ readings were performed between 11:00 and 16:00 h on three leaves per plant in each treatment and the values were averaged (Chavez-Arias et al., 2020).

#### 2.6.2 Leaf temperature, relative chlorophyll content, and chlorophyll α fluorescence parameters

Leaf temperature, relative chlorophyll content, and chlorophyll α fluorescence parameters (variable-to-maximum chlorophyll fluorescence ratio (F_v_/F_m_) and non-photochemical chlorophyll fluorescence quenching (NPQ)) were measured using the MultispeQ v1 instrument (Kuhlgert et al., 2016) at the end of each experiment. The readings were taken according to the protocol “Photosynthesis leaf MultispeQ v2.0”. A sensor in the MultispeQ detects ambient photosynthetically active radiation (PAR) in the field and reproduces this PAR value using an internal LED. When the sensor is placed on the leaf, the reading begins by recording local PAR, leaf temperature, ambient temperature, and leaf angle and position. The first set of optical measurements was recorded after a brief period of illumination in the measured ambient PAR. Once completed, the leaf is exposed to a period of high PAR (2,000 μmol · m^−2^ s^−1^, equivalent to full sun) for 10 s. Optical measurements were repeated at high PAR. The leaf is then dark-adapted (by turning off the actinic light), with a weak far-red backlight for 10 s. A final set of optical measurements was performed to assess the rapid dissipation of NPQ. The reading on a single leaf took approximately 120 seconds. The readings were taken on the same leaves that were used for g_s_ estimation.

#### 2.6.3 pH, electrical conductivity, and concentrations of nitrate, calcium, and potassium in the sap

At the end of the experiments (55 DAE for experiment 1 and 88 DAE for experiment 2), eight mature rice leaf sheaths were collected from each treatment for the first experiment, whereas the flag leaf sheath was used for the second experiment. The methodology described by Grijalva-Contreras et al. (2016) was adapted for these experiments. The sap was extracted using a mechanical press and four drops of sap were placed in the respective ionometers (Horiba, Kyoto, JA) to determine the PH, electrical conductivity (mS cm^-1^), and concentrations of nitrate (NO_3_ in mg L^- 1^), calcium (Ca^++^ in mg L^-1^) and potassium (K^+^ in mg L^-1^). Finally, five readings were recorded per treatment.

#### 2.6.4 Crop Stress Index (CSI) and Relative Tolerance Index (RTI)

The calculation of the CSI used the methodology described by Gerhards et al. (2016) in both experiments. Photographs were taken with a thermal camera (FLIR 2, FLIR Systems Inc., MA, US) with an accuracy of ± 2°C at the end of the stress period of each of the experiments. A white surface was placed behind the plants for photography. Two plants were used as the reference standard. These plants were placed on a white surface; one of them was covered with an agricultural adjuvant (Agrotin, Bayer CropScience, Colombia) to simulate the total stomatal opening (wet pattern (Twet)), and the other was a plant without any application (dry pattern (Tdry)) (Castro-Duque et al., 2020). The photograph was taken considering 1 m between the camera and the pot. With the photographs obtained, the CSI was calculated using the following equation (1).


Equation 1 $$CSI=\frac{Tleaf\;-\;Twet\;}{Tdry\;-\;Twet\;}$$

The RTI was calculated indirectly to determine the tolerance of the treated genotypes evaluated in this research, using g_s_ of plants with combined heat stress compared to control plants (plants without stress treatment or application of growth regulators). RTI was obtained using equation (2) adapted from Chávez-Arias et al. (2020).


Equation 2 $$RTI=\left(g_{s}plantsunderthermalstress/g_{s}plantswithoutthermalstress\right)*100$$

All physiological variables described above were determined and recorded at 55 DAE in the first experiment, using fully expanded leaves collected from the upper part of the canopy. For the second experiment, the variables described above were determined and recorded at 80 (start of stress), 88 (end of stress), and 96 DAE (recovery period) using the flag leaf of each plant. The measurements were taken inside the growth chamber to avoid altering the environmental conditions of the plants in both tests.

#### 2.6.5 Biochemical variables: Leaf photosynthetic pigments, lipid peroxidation (malondialdehyde - MDA), and proline

Approximately 800 mg of fresh weight of leaves was collected for the biochemical variables in each of the experiments at 55 DAE for E1 and 88 DAE for E2. The leaf samples were then homogenized in liquid nitrogen and stored for later analysis. The spectral determination method used to estimate the content of chlorophyll a, b and carotenoids in the tissue was based on the methodology and equations described by Wellburn (1994). Leaf tissue samples (30 mg) were collected and homogenized in 3 mL of 80% acetone. The samples were then centrifuged (Model 420101, Becton Dickinson Primary Care Diagnostics, US) at 5000 rpm for 10 min to remove particles. The supernatant was diluted to a final volume of 6 ml by adding 80% acetone (Sims and Gamon, 2002). Chlorophyll content was determined at 663 (chlorophyll a) and 646 (chlorophyll b) nm, while carotenoids were estimated at 470 nm using a spectrophotometer (Spectronic BioMate 3 UV-vis, Thermo, USA).

The thiobarbituric acid (TBA) method described by Hodges et al. (1999) was used to estimate membrane lipid peroxidation (MDA). Approximately 0.3 g of leaf tissue was also homogenized in liquid nitrogen. The samples were centrifuged at 5000 rpm and then the absorbances were measured at 440, 532, and 600 nm with the spectrophotometer. Finally, an extinction coefficient (157 M·ml^−1^) was used to obtain the MDA concentration.

For all treatments, proline content was determined using the method described by Bates et al. (1973). Ten ml of a 3% aqueous solution of sulfosalicylic acid was added to the stored samples, which were then filtered through Whatman paper (No. 2). Subsequently, 2 mL of this filtrate was reacted with 2 mL of ninhydrin acid and 2 mL of glacial acetic acid. The mixture was placed in a water bath at 90°C for 1 h. The reaction was stopped by incubation on ice. The resulting solution was dissolved in 4 ml of toluene by shaking the test tubes vigorously using a vortex mixer. Absorbance readings were determined at 520 nm with the same spectrophotometer used in the quantification of photosynthetic pigments (Spectronic BioMate 3 UV-Vis, Thermo, Madison, USA).

### 2.7 Variables determined in E3

The following physiological variables and panicle characteristics were determined in E3:

#### 2.7.1 Photosynthetic variables: Photosynthesis (P_n_), transpiration (*E*), stomatal conductance (g_s_), intercellular carbon (C_i_), water use efficiency (WUE), carboxylation efficiency (P_n_/C_i_), F_v_/F_m_ ratio, and non-photochemical quenching (NPQ)

The methodology described by Alvarado-Sanabria et al. (2017) was used to measure the leaf photosynthetic variables. The gas exchange readings were taken at the end of the stress period to which the plants were subjected (88 DAE for the plants at phenological stage R2 and 108 DAE for the plants at phenological stage R8) using a portable infrared gas analyzer (LI-6400XT; LI-COR, Lincoln, NE) between 10:00 a.m. and 3:00 p.m. The flag leaf of the plant was selected to perform the readings of these variables. The leaves were previously adapted to the surrounding environment without touching their surface to avoid stomatal closure before the measurements. The variables measured were the following: Photosynthesis (P_n_), stomatal conductance (g_s_), transpiration (*E*), and intercellular CO_2_ concentration inside the leaf (C_i_). During the readings, the LI-COR chamber conditions were as follows: A red:blue light source (model 6400-02B LED; LICOR) provided 1200 µmol m^−2^ s^−1^ PPFD, which is the light saturation determined with a light response curve performed before the beginning of the experiment. Other settings included a reference CO_2_ level of 400 mmol mol^–1^, a constant chamber temperature of 27°C, and a gas flow rate of 400 mmol s^–1^. The intrinsic water use efficiency (WUE) was calculated by the P_n_/g_s_ ratio and the carboxylation efficiency (P_n_/C_i_) (Flexas et al., 2001).

Chlorophyll α fluorescence parameters (ratio of variable to maximum chlorophyll fluorescence (F_v_/F_m_) and non-photochemical chlorophyll fluorescence quenching (NPQ) were measured using the MultispeQ v1 instrument (Kuhlgert et al., 2016) at end of each stress period (88 DAE for plants at phenological stage R2; 108 DAE for plants at phenological stage R8) using the methodology described in E2.

#### 2.7.2 Characteristics of the panicle

The panicles were harvested at the end of the growth cycle of the genotype F2000 (120 DAE). Then, the following characteristics of the panicle were determined: filled spikelets, blank spikelets, total spikelets, and percentage of panicle blanking.

### 2.8 Experimental design and statistical analysis

Data from the three different experiments were analyzed separately. A completely randomized design was used for E1 and E2, respectively, whereas a factorial arrangement (the main factor was the phenological stages and the secondary factor was the foliar application treatments) was used in E3. Each treatment group consisted of five plants and each plant was an experimental unit in all experiments. An analysis of variance (ANOVA) (P ≤ 0.05) was performed in each experiment. When significant differences were found, Tukey’s *post hoc* comparative test was used at P ≤ 0.05. Percentage values were transformed using the arcsine function. Data were analyzed using the Statistix v 9.0 software (Analytical Software, Tallahassee, FL, USA) and SigmaPlot (version 10.0; Systat Software, San Jose, CA, USA) was used for the figures.

## 3 Results

### 3.1 First experiment (E1): Evaluation of foliar sprays of cytokinins or brassinosteroids in rice seedlings

#### 3.1.1 Stomatal conductance and leaf temperature

**Figure 1:**
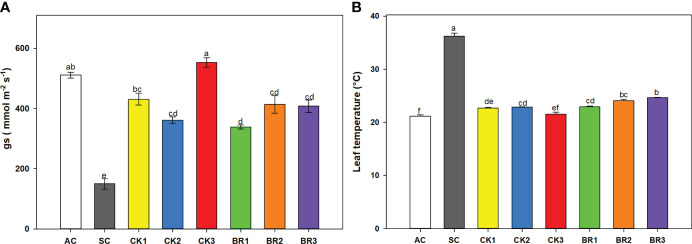
Effect of the number of applications of two growth regulators (cytokinins (CK) or brassinosteroids (BR)) on stomatal conductance (g_s_) **(A)** and leaf temperature **(B)** in rice plants of genotype Fedearroz 2000 subjected to combined heat stress (40°C day/30°C night) at 55 days after emergence (DAE). The treatments evaluated were as follows: absolute control (AC), heat stress control (SC), one foliar spray of CK (CK1), two foliar sprays of CK (CK2), three foliar sprays of CK (CK3), one spray foliar spray of BR (BR1), two foliar sprays of BR (BR2) or three foliar sprays of BR (BR3). Each column represents the mean of five data ± standard error (n = 5). Bars followed by different letters indicate statistically significant differences according to Tukey’s test (P ≤ 0.05). Equal letters indicate that the means are not statistically significant (≤ 0.05).

Differences in g_s_ (P ≤ 0.01) were found between treatments (**Figure 1A**). Rice seedlings subjected only to combined heat stress (SC) showed the lowest g_s_ values (149.5 mmol m^-2^ s^-1^) compared to the absolute control (AC) (511 mmol m^-2^ s^-1^). Rice plants treated with a different number of foliar applications of cytokinins (CK) or brassinosteroids (BR) and subjected to combined heat stress registered values higher than those of plants subjected to SC (between 400 and 552.85 mmol m^-2^ s^-1^ in all treatments, with the highest value for CK3 (552.85 mmol m^-2^ s^-1^)). Leaf temperature also showed significant differences between treatments (P ≤ 0.01) (**Figure 1B**). Opposite trends were found for this variable. The SC treatment registered the highest temperature of all the treatments (36.21°C). The temperatures recorded for plants with different numbers of foliar applications of CK were between 21°C and 23°C, whereas for BR temperatures ranged between 23°C and 25°C. In these cases, the values were lower than those recorded in the SC treatment. Finally, the leaf temperatures observed in the rice plants treated with CK or BR were close to the values recorded by the AC (21.16°C).

#### 3.1.2 F_v_/F_m_ ratio, non-photochemical quenching (NPQ), and chlorophyll content

**Figure 2:**
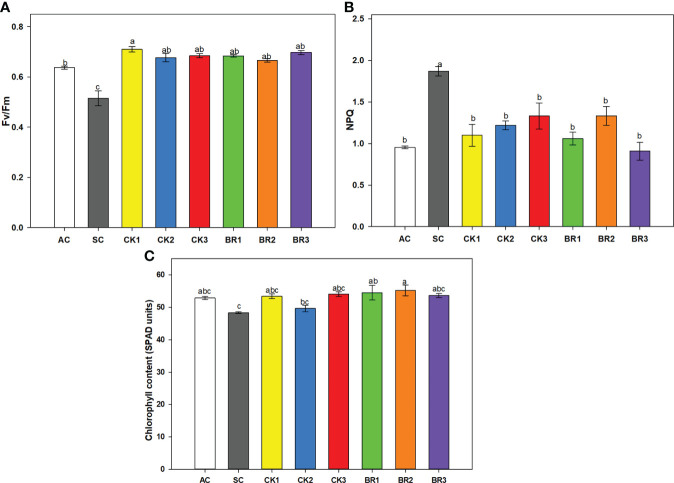
Effect of the number of sprays of two growth regulators (cytokinins (CK) or brassinosteroids (BR)) on the ratio of variable to maximum chlorophyll fluorescence from photosystem II (F_v_/F_m_) **(A)**, non-photochemical quenching (NPQ) **(B)**, and chlorophyll content **(C)** in rice plants of genotype Fedearroz 2000 subjected to combined heat stress (40°C day/30°C night) at 55 days after emergence (DAE). The treatments evaluated were as follows: absolute control (AC), heat stress control (SC), one foliar spray of CK (CK1), two foliar sprays of CK (CK2), three foliar sprays of CK (CK3), one foliar spray of BR (BR1), two foliar sprays of BR (BR2) or three foliar sprays of BR (BR3). Each column represents the mean of five data ± standard error (n = 5). Bars followed by different letters indicate statistically significant differences according to Tukey’s test (P ≤ 0.05). Equal letters indicate that the means are not statistically significant (≤ 0.05).

The F_v_/F_m_ ratio showed significant differences between treatments (P ≤ 0.01) as observed in **Figure 2A**. Stressed plants without any growth regulator sprays showed the lowest F_v_/F_m_ ratio (0.51). However, the different foliar applications of CK or BR helped to improve this variable, increasing this ratio between 0.67 and 0.71 for CK and 0.66 and 0.70 for BR. The values of the treatments were higher than those of the AC (0.63). Non-photochemical quenching (NPQ) showed opposite trends to the F_v_/F_m_ ratio, where the highest value was observed in plants of the SC treatment. Additionally, there were no significant differences between the rice plants treated with CK or BR and the AC (0.95) (**Figure 2B**). Finally, the chlorophyll content (SPAD units) (**Figure 2C**) was also decreased by the effect of combined heat stress in the SC treatment, registering values of 48.34. The different number of foliar applications with CK or BR favored the relative content of this pigment in rice plants under combined heat stress (53.38 for CK1, 49.62 for CK2, 54.05 for CK3, 54.50 for BR1, 55.22 for BR2, and 53.61 for BR3). The readings obtained from the BR or CK treatments were similar to those obtained in plants grown under normal conditions (AC) (52.86).

#### 3.1.3 pH, electrical conductivity, concentration of nitrate, calcium, and potassium in the sap

**Figure 3:**
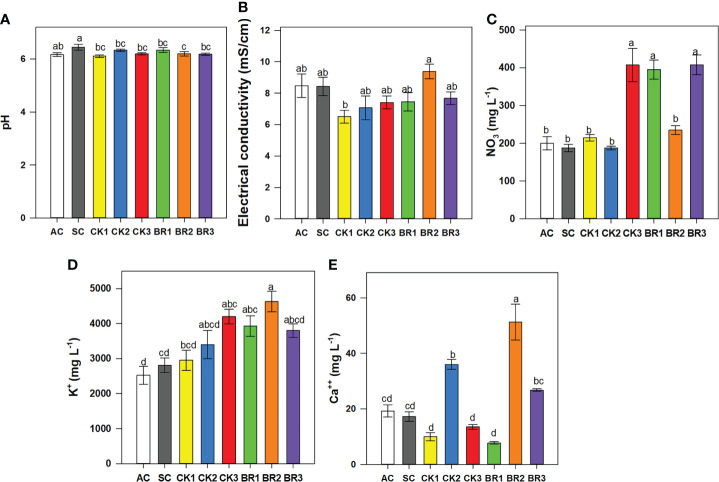
Effect of the number of applications of two growth regulators (cytokinins (CK) or brassinosteroids (BR)) on pH **(A)**, electrical conductivity **(B)**, and contents of nitrate **(C)**, potassium **(D)**, and calcium **(E)** in rice plants of genotype Fedearroz 2000 subjected to combined heat stress (40°C day/30°C night) at 55 days after emergence (DAE). The treatments evaluated were as follows: absolute control (AC), heat stress control (SC), one foliar spray of CK (CK1), two foliar sprays of CK (CK2), three foliar sprays of CK (CK3), one foliar spray of BR (BR1), two foliar sprays of BR (BR2) or three foliar sprays of BR (BR3). Each column represents the mean of five data ± standard error (n = 5). Bars followed by different letters indicate statistically significant differences according to Tukey’s test (P ≤ 0.05). Equal letters indicate that the means are not statistically significant (≤ 0.05).

The pH also showed significant differences between treatments (P ≤ 0.01) (**Figure 3A**). The treatment that was under combined heat stress and without any sprays of foliar growth regulators (SC: 6.44) showed slightly higher values than the rest of the treatments (AC: 6.14 and the different foliar applications of CK or BR: between 5.8 and 5.99). The electrical conductivity registered a different behavior compared to the pH. The highest value of electrical conductivity was obtained in plants treated twice with BR (BR2: 9.37 mS cm^-1^), whereas the CK1 treatment (stressed plants with two CK sprays) recorded the lowest values (6.51 mS cm^-1^). There were no significant differences between the other treatments (values ranged from 7 mS cm^-1^ to 8.5 mS cm^-1^) (**Figure 3B**).

Regarding the concentration of nutrients in sap, nitrate (mg L^-1^) showed significant differences (P ≤ 0.01) between treatments (**Figure 3C**). The rice plants of CK3, BR1, or BR3 registered higher values between treatments (407.5, 395, and 407.5 mg L^-1^, respectively) followed by the rest of the treatments including AC and SC (readings between 185 and 240 mg L^-1^). The potassium content was lower in plants grown under normal conditions (AC) (2525 mg L^-1^), whereas intermediate readings were observed in rice plants under heat stress and without foliar sprays (SC) (2812 mg L^-1^). Rice plants treated with different foliar applications of CK or BR were between 2950 and 4625 mg L^-1^, being the BR2 treatment the one that showed the highest readings (4625 mg L^-1^) for this cation (**Figure 3D**). Finally, the treatments CK2, BR2, and BR3 improved the concentration of calcium within the plant (36, 51.25, and 26.75 mg L^-1^, respectively) (**Figure 3E**). For the rest of the treatments, the registered values were between 7.75 and 19.25 mg L^-1^, with the last value for the AC treatment.

#### 3.1.4 Crop Stress Index (CSI) and Relative Tolerance Index (RTI)

The effect of the different number of foliar sprays of growth regulators (BR or CK) and the combined heat stress on the relative tolerance index (RTI) and crop stress index (CSI) is shown in **Table 1**. For CSI, differences were observed between the different treatments of rice plants under the stress condition (P ≤ 0.01). Rice plants only exposed to combined heat stress showed the highest CSI values (0.95). The CSI was lower (values between 0.1 and 0.45) when the rice plants were treated with the different numbers of sprays of plant growth regulators. Finally, rice plants grown under optimal conditions showed a value of 0.38. On the other hand, the RTI showed a behavior similar to that of the rest of the physiological variables, with significant differences (P ≤ 0.01) being observed between treatments. SC plants showed lower tolerance than the rest of the treatments (29.25%). Foliar application of plant hormones improved RTI in plants exposed to stressful temperatures. This effect was more noticeable in the ‘F2000’ plants treated with three sprays of CK (RTI of 108%) and with two or three sprays of BR (RTI of 100% and 98.53%, respectively).

**Table 1:** Effect of the number of applications of two growth regulators (cytokinins (CK) or brassinosteroids (BR)) on the crop stress index (CSI) and relative tolerance index (RTI) of rice plants of genotype F2000 subjected to combined heat stress (40°C day/30°C night) at 55 days after emergence (DAE).

Treatments	CSI	RTI (%)
Absolute control	0.38 b	——
Heat stress control	0.95 a	29.25 d
One spray of CK	0.23 b	84.27 bc
Two sprays of CK	0.29 b	70.72 c
Three sprays of CK	0.19 b	108.18 a
One spray of BR	0.30 b	66.23 c
Two sprays of BR	0.40 b	100 ab
Three sprays of BR	0.41 b	98.53 ab
Significance	***	***

The treatments evaluated were as follows: absolute control (AC), heat stress control (SC), one foliar spray of CK (CK1), two foliar sprays of CK (CK2), three foliar sprays of CK (CK3), one foliar spray of BR (BR1), two foliar sprays of BR (BR2) or three foliar sprays of BR (BR3). Data represent the mean of five data points ± standard error (n = 5). Data followed by different letters indicate statistically significant differences according to Tukey’s test (P ≤ 0.05). Equal letters indicate that the means are not statistically significant (≤0.05). NS, *, ** or *** not significant or significant at P ≤ 0.05, 0.01 or 0.001, respectively.

#### 3.1.5 Biochemical variables: Foliar photosynthetic pigments, lipid peroxidation (malondialdehyde - MDA), and proline

The differences (P ≤ 0.01) between the treatments on the foliar content of photosynthetic pigments (chlorophyll and carotenoids) are shown in **Table 2**. High temperatures (day/night) caused the degradation of the total contents of chlorophyll and carotenoids. Rice seedlings without foliar applications of plant hormones and exposed to combined heat stress showed a drop in the contents of chlorophyll a and b (2.13 mg g^-1^ for chlorophyll a and 0.67 mg g^-1^ for chlorophyll b) compared to plants grown under optimal temperature conditions (2.24 mg g^-1^ for chlorophyll a and 0.75 mg g^-1^ for chlorophyll b). In general, foliar applications of CK or BR favored chlorophyll synthesis under stress conditions, where foliar treatments of two or three sprays of CK or BR caused an increase in this variable under heat stress conditions (2.0 mg g^-1^ to 3. 93 mg g^-1^).

**Table 2:** Effect of the number of sprays of two growth regulators (cytokinins (CK) or brassinosteroids (BR)) on biochemical variables of rice plants of genotype Fedearroz 2000 subjected to combined heat stress (40°C day/30°C night) at 55 days after emergence (DAE).

Treatments	Chl amg g^-1^ (FW)	Chl bmg g^-1^ (FW)	Total Chl mg g^-1^ (FW)	Cx+cmg g^-1^ (FW)	MDA µmol g^-1^ (FW)	Proline µmol g^-1^ (FW)
Absolute control	2.24 ab	0.75 ab	2.99 ab	0.43 cd	4.38 b	1.37 c
Heatstress control	2.13 bc	0.67 bc	2.80 bc	0.50 abc	16.12 a	0.91 c
One spray of CK	1.49 e	0.59 cd	2.07 d	0.28 f	6.36 b	11.63 ab
Two sprays of CK	1.58 de	0.54 d	2.12 d	0.34 ef	6.77 b	4.10 bc
Three sprays of CK	2.57 a	0.81 a	3.39 a	0.58 a	3.02 b	5.69 bc
One spray of BR	1.71 de	0.59 cd	2.29 d	0.37 de	7.65 b	13.14 ab
Two sprays of BR	2.27 ab	0.73 ab	3.00 ab	0.52 ab	4.14 b	16.90 a
Three sprays of BR	2.10 bc	0.66 bc	2.76 bc	0.48 bc	3.80 b	18.86 a
Significance	***	***	***	***	***	***

The treatments evaluated were as follows: absolute control (AC), heat stress control (SC), one foliar spray of CK (CK1), two foliar sprays of CK (CK2), three foliar sprays of CK (CK3), one foliar spray of BR (BR1), two foliar sprays of BR (BR2) or three foliar sprays of BR (BR3). Data represent the mean of five data points ± standard error (n = 5). Data followed by different letters indicate statistically significant differences according to Tukey’s test (P ≤ 0.05). Equal letters indicate that the means are not statistically significant (≤0.05). NS, *, ** or *** not significant or significant at P ≤ 0.05, 0.01 or 0.001, respectively.

The contents of MDA (P ≤ 0.01) and proline (P ≤ 0.01) also showed significant differences between the different treatments (**Table 2**). Rice seedlings without foliar applications of plant hormones and exposed to a short period of combined heat stress (40°C day/30°C night) also showed higher MDA synthesis (16.12 µmol g^-1^ (FW)) compared to plants grown under optimal temperature conditions or stressed plants treated with the different numbers of growth regulator sprays (values between 4.38 µmol g^-1^ and 7.7 µmol g^-1^ (FW) for the rest of the treatments). In general, CK or BR sprays at different times of application reduced the production of MDA (registering values between 3.02 µmol g^-1^ (FW) and 7.70 µmol g^-1^ (FW) for the content of MDA) under stress conditions. Regarding proline content, rice seedlings without foliar applications of plant hormones showed a lower proline content (0.91 µmol g^-1^ (FW)) compared to plants grown under optimal temperature conditions (1.37 µmol g^-1^ (FW)) and the rest of the treatments in the experiment. Rice seedlings subjected to heat stress and treated with one, two, or three sprays of CK showed an increase in proline content with values between 4.00 and 12.00 µmol g^-1^ (FW). Foliar treatments of one or two sprays of BR caused an increase in proline content under heat stress conditions (values of 13.14 µmol g^-1^ (FW) and 16.90 µmol g^-1^ (FW), respectively). The foliar treatment of three sprays of BR showed the highest proline synthesis among all treatments (18.86 µmol g^-1^ (FW)).

### 3.2 Second experiment (E2): Effect of foliar sprays of cytokinins or brassinosteroids on the flag leaves of rice plants

#### 3.2.1 Stomatal conductance and leaf temperature

**Figure 4:**
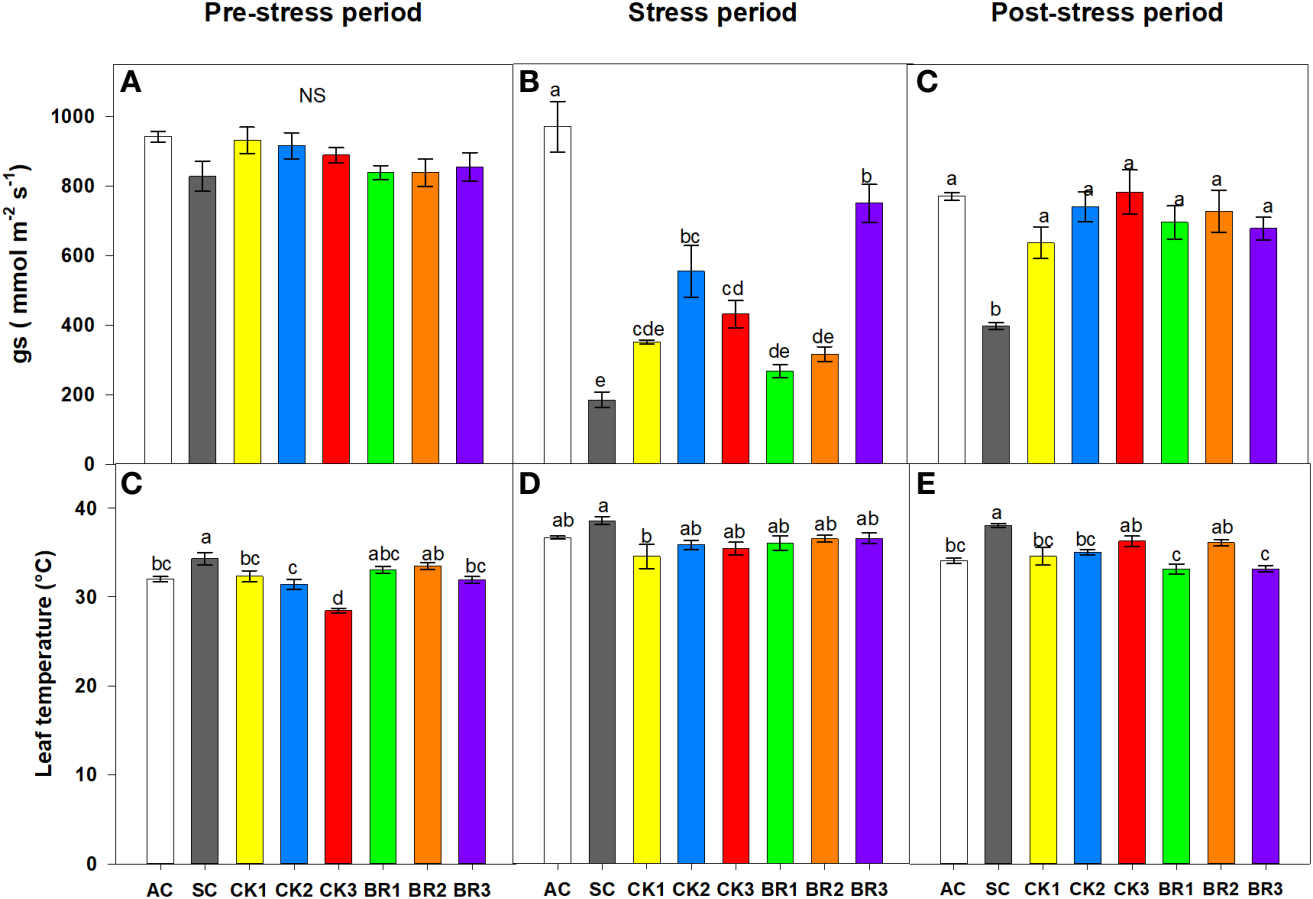
Effect of the number of applications of two growth regulators (cytokinins (CK) or brassinosteroids (BR)) on stomatal conductance (g_s_) before the stress period **(A)**, end of the stress period **(B)**, and after the stress period (recovery) **(C)**, and leaf temperature before the stress period **(D)**, during the stress period **(E)**, and after the stress period (recovery) **(F)** in rice plants of genotype F2000 subjected to combined heat stress (40°C day/30°C night) at 88 days after emergence (DAE). The treatments evaluated were as follows: absolute control (AC), heat stress control (SC), one foliar spray of CK (CK1), two foliar sprays of CK (CK2), three foliar sprays of CK (CK3), one foliar spray of BR (BR1), two foliar sprays of BR (BR2) and three foliar sprays of BR (BR3). Each column represents the mean of five data ± standard error (n = 5). Bars followed by different letters indicate statistically significant differences according to Tukey’s test (P ≤ 0.05). Equal letters indicate that the means are not statistically significant (≤ 0.05).

In this experiment, g_s_ and leaf temperature were recorded before the combined heat stress period, during the stress period, and eight days after the stress period (recovery). For g_s,_ no significant differences were found between treatments before the stress period (**Figure 4A**). During the stress period, rice plants only subjected to combined heat stress (SC) showed the lowest g_s_ values (149.5 mmol m^-2^ s^-1^). On the other hand, rice plants treated with different numbers of CK applications recorded values between 350 and 555 mmol m^-2^ s^-1^, and between 260 and 750 mmol m^-2^ s^-1^ for BR applications. However, treatments with both hormones substantially improved g_s_, but these values did not match those obtained in plants grown under normal conditions (AC: 970.4 mmol m^-2^ s^-1^) (**Figure 4B**). After the recovery period, rice plants treated with a different number of foliar sprays of either CK or BR did not show significant differences compared to AC. Finally, the flag leaves of the SC treatment continued to show the lowest values of all the treatments (397.75 mmol m^-2^ s^-1^) (**Figure 4C**).

Leaf temperature also showed significant differences between treatments (P ≤ 0.01) during the three different measurement moments (**Figures 4D–F**). Before the beginning of the combined period of heat stress, the CK3 treatment obtained the lowest temperature among all the treatments (28.4°C), whereas rice plants of the SC registered one of the highest temperatures (34.3°C). The values of the other treatments (CK1, CK2, BR1, BR2, and BR3) ranged between 31°C and 33.5°C. On the other hand, the leaf temperature of the flag leaves of the SC treatment continued to be higher (37.1°C) during the stress period. CK or BR sprays caused a reduction in temperature (ranging from 34°C to 37°C), observing that this group of plants showed values close to those recorded in unstressed and untreated rice plants. (AC: 31.16°C). Eight days after the stress period (recovery period), rice plants treated with a different number of foliar sprays of CK or BR registered values between 33°C and 36°C, similar to those obtained in the flag leaves of AC plants (34°C). Finally, the SC treatment continued to have the highest leaf temperature (38°C) with no variation of this variable being observed in the recovery period compared to the end of the period of exposure to the combined heat stress.

#### 3.2.2 F_v_/F_m_ ratio, non-photochemical quenching (NPQ), and chlorophyll content

**Figure 5:**
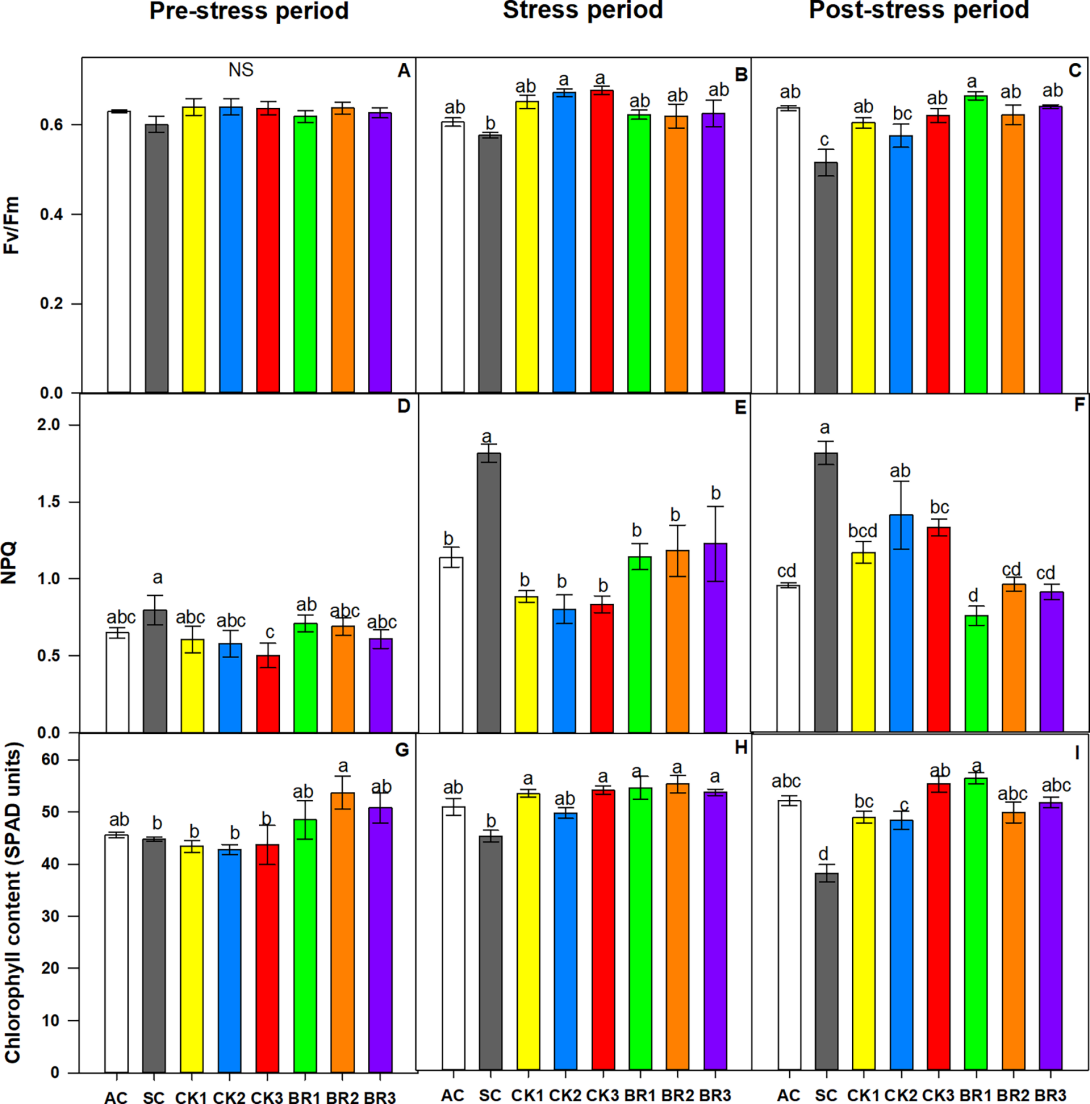
Effect of the number of applications of two growth regulators (cytokinins (CK) or brassinosteroids (BR)) on the ratio of variable to maximum chlorophyll fluorescence of photosystem II (F_v_/F_m_) before the stress period **(A)**, during the stress period **(B)**, and after the stress period (recovery) **(C)**, non-photochemical quenching (NPQ) before the stress period **(D)**, during the stress period **(E)**, after the stress period **(F)**, and chlorophyll content before the stress period **(G)**, during the stress period **(H)**, and after the stress period **(I)** in rice plants of genotype F2000 subjected to combined heat stress (40°C day/30°C night) at 88 days after emergence (DAE). The treatments evaluated were as follows: absolute control (AC), heat stress control (SC), one foliar spray of CK (CK1), two foliar sprays of CK (CK2), three foliar sprays of CK (CK3), one foliar spray of BR (BR1), two foliar sprays of BR (BR2), and three foliar sprays of BR (BR3). Each column represents the mean of five data ± standard error (n = 5). Bars followed by different letters indicate statistically significant differences according to Tukey’s test (P ≤ 0.05). Equal letters indicate that the means are not statistically significant (≤ 0.05).

Regarding the F_v_/F_m_ ratio, no significant differences were found between treatments before the stress period (**Figure 5A**). During the stress period, rice plants only subjected to combined heat stress (SC) showed the lowest F_v_/F_m_ ratio (0.57) (**Figure 5B**). Rice plants treated with BR registered values between 0.61 and 0.63, whereas plants treated with CK showed values between 0.65 and 0.68. The highest value of this variable was recorded in plants treated with CK2 or CK3 (0.671 and 0.767, respectively), whereas the value obtained for the absolute control (AC) was 0.60. Eight days after the stress period, most of the rice plants treated with a different number of foliar sprays of CK or BR did not show significant differences compared to the AC (± 0.65). The SC treatment continued to show the lowest value of all treatments with a ratio of 0.51.

The non-photochemical quenching (NPQ) also showed significant differences between treatments (P ≤ 0.01) during the three measurement moments. Before the stress period, plants subjected to high temperatures and treated with three foliar applications of CK obtained the lowest NPQ (0.50), whereas the plants exposed to stress (SC) showed the highest coefficient (0.79). The plants subjected to stress and treated with BR registered NPQ values between 0.57 and 0.70. At the end of the combined heat stress period, the SC treatment continued to show the highest NPQ value (1.81) (**Figure 5E**). There were no significant differences between the rest of the treatments regarding said coefficient (values between 0.80 and 1.22), indicating that foliar application helped this variable under stress conditions. Eight days after the stress period, the rice plants treated with a different number of foliar sprays of CK or BR registered values between 1 and 1.5, showing similar results to those of the AC plants (1.2). The SC treatment continued to show the highest value of all treatments with a temperature of 1.8 (**Figure 5F**).

The relative content of chlorophyll (SPAD units) also showed differences (P ≤ 0.01) due to the different treatments during the three measurement moments (**Figures 5G, H, I**). Before the combined heat stress period, rice plants treated with a different number of foliar sprays of BR showed a higher chlorophyll content than the rest of the treatments (48.4 for BR1, 53.7 for BR2, and 50.7 for BR3). For the rest of the treatments, the chlorophyll content values were between 42 and 45 SPAD units. During the combined stress period, the SC treatment also showed the lowest chlorophyll content (45.89). Plants treated with the different applications of growth regulators (CK or BR) showed an increase in the content of chlorophylls, possibly due to an increase in the synthesis of these compounds. The AC and CK2 treatments showed values of 50.8 and 49.6 SPAD units, respectively, whereas CK3, BR1, BR2, or BR3 recorded the highest values (ranging between 53.5 and 55 SPAD units). Eight days after the stress period, rice plants treated with a different number of foliar sprays of CK or BR continued to show higher chlorophyll content values than the SC treatment (≥ 48 SPAD units) but did not reach the values observed in the AC (51 SPAD units).

#### 3.2.3 pH, electrical conductivity, and concentration of nitrate, calcium, and potassium in the sap

**Figure 6:**
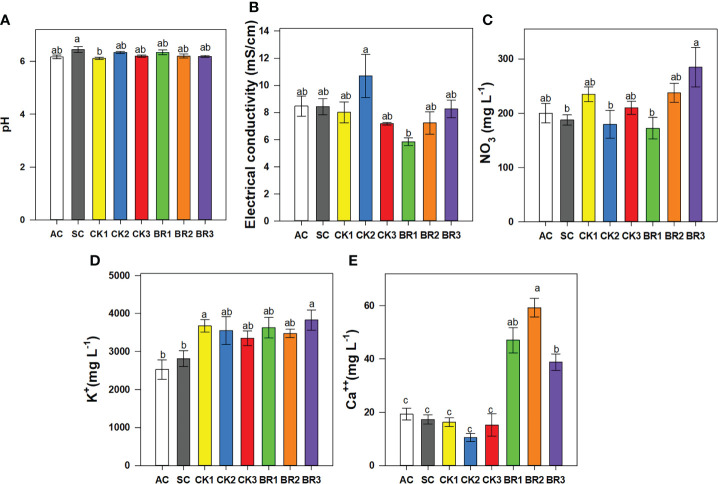
Effect of the number of applications of two growth regulators (cytokinins (CK) or brassinosteroids (BR)) on the pH **(A)**, electrical conductivity **(B)**, contents of nitrate **(C)**, potassium **(D)**, and calcium **(E)** in rice plants of genotype F2000 subjected to combined heat stress (40°C day/30°C night) at 88 days after emergence (DAE). The treatments evaluated were as follows: absolute control (AC), heat stress control (SC), one foliar spray of CK (CK1), two foliar sprays of CK (CK2), three foliar sprays of CK (CK3), one foliar spray of BR (BR1), two foliar sprays of BR (BR2) and three foliar sprays of BR (BR3). Each column represents the mean of five data ± standard error (n = 5). Bars followed by different letters indicate statistically significant differences according to Tukey’s test (P ≤ 0.05). Equal letters indicate that the means are not statistically significant (≤ 0.05).

The pH showed significant differences between treatments (P ≤ 0.01) as observed in **Figure 6A**. Rice plants under combined heat stress and without any application of foliar growth regulators showed a more basic pH compared to the other treatment groups (6.45). Stressed plants with foliar applications of CK showed a lower pH (6.10), whereas the remaining groups of plants (CK, BR, or AC) obtained intermediate values (between 6.16 and 6.33). The electrical conductivity showed significant differences between treatments (P ≤ 0.01) (**Figure 6B**). Rice plants under combined heat stress and without any applications of foliar growth regulators did not show differences in their electrical conductivity when compared to the AC. Stressed plants with a foliar application of CK showed a higher electrical conductivity (11.5 mS cm^-1^), whereas the remaining groups of plants (CK, BR, or AC) did not show significant differences between them, registering values from 7.17 mS cm^-1^ and 8.5 mS cm^-1^.

Differences were observed in the concentration of nitrate, potassium, and calcium in rice plants due to the different treatment groups (**Figures 6C–E**). In general, AC and SC plants showed the lowest contents of nitrate (200 mg L^-1^ and 180 mg L^-1^, respectively), potassium (2500 mg L^-1^ and 2800 mg L^-1^, respectively), and calcium (20 mg L^-1^ and 18 mg L^-1^, respectively). Plants with different numbers of CK applications showed contents of nitrate between 180 mg L^-1^ and 280 mg L^-1^, potassium between 3300 mg L^-1^ and 3800 mg L^-1^, and calcium between 10 mg L^-1^ and 15 mg L^-1^. Regarding the different BR applications, we observed contents of nitrate between 180 mg L^-1^ and 250 mg L^-1^, potassium between 3200 mg L^-1^ and 3500 mg L^-1^, and calcium between 35 mg L^-1^ and 50 mg L^-1^.

#### 3.2.4 Biochemical variables: Leaf photosynthetic pigments, lipid peroxidation (malondialdehyde - MDA), and proline

Differences (P ≤ 0.01) in photosynthetic pigments of flag leaves between treatments are shown in **Table 3**. High day/night temperatures caused higher total chlorophyll and carotenoid content. Rice seedlings without foliar sprays of plant hormones showed higher chlorophyll a (2.22 mg g^-1^) and b (0.82 mg g^-1^) contents compared to plants grown under optimal temperature conditions (1.94 mg g^-1^ for chlorophyll a and 0.80 mg g^-1^ for chlorophyll b). Foliar treatments of two or three sprays of BR caused an increase in this variable under conditions of heat stress (values of 2.40 mg g^-1^ and 2.23 mg g^-1^ for chlorophyll a and values between 0.94 mg g^-1^ and 0.87 mg g^-1^ for chlorophyll b). The foliar treatment of three CK applications favored the highest synthesis of chlorophyll under stressful conditions due to the higher values of photosynthetic pigment compared to the rest of the treatments (2.67 mg g^-1^ for chlorophyll a and 0.96 mg g^-1^ for chlorophyll b).

**Table 3:** Effect of the number of applications of two growth regulators (cytokinins (CK) or brassinosteroids (BR)) on biochemical variables in rice plants of genotype F2000 subjected to combined heat stress (40°C day/30°C night) at 88 days after emergence (DAE).

Treatments	Chl amg g^-1^ (FW)	Chl bmg g^-1^ (FW)	Total Chl mg g^-1^ (FW)	Cx+cmg g^-1^ (FW)	MDA µmol g^-1^ (FW)	Proline µmol g^-1^ (FW)
Absolute control	2.54 ab	0.80 ab	3.34 ab	0.40 bc	4.45 b	2.11 c
Heat stress control	2.22 bc	0.82 bc	3.04 bc	0.47 ab	16.19 a	1.49 c
One spray of CK	1.58 e	0.74 cd	2.32 d	0.25 e	6.43 b	12.48 ab
Two sprays of CK	1.68 de	0.70 d	2.37 d	0.31 de	6.84 b	4.65 bc
Three sprays of CK	2.67 a	0.96 a	3.63 a	0.55 a	3.09 b	6.33 bc
One spray of BR	1.81 d	0.75 cd	2.55 d	0.34 cd	7.72 b	13.78 ab
Two sprays of BR	2.40 ab	0.94 a	3.35 ab	0.47 ab	4.21 b	17.81 a
Three sprays of BR	2.23 bc	0.87 ab	3.10 bc	0.44 b	3.87 b	19.43 a
Significance	***	***	***	***	***	***

The treatments evaluated were as follows: absolute control (AC), heat stress control (SC), one foliar spray of CK (CK1), two foliar sprays of CK (CK2), three foliar sprays of CK (CK3), one foliar spray of BR (BR1), two foliar sprays of BR (BR2) and three foliar sprays of BR (BR3). Data represent the mean of five data points ± standard error (n = 5). Data followed by different letters indicate statistically significant differences according to Tukey’s test (P ≤ 0.05). Equal letters indicate that the means are not statistically significant (≤0.05). NS, *, ** or *** not significant or significant at P ≤ 0.05, 0.01 or 0.001, respectively.

The contents of MDA (P ≤ 0.01) and proline (P ≤ 0.01) also showed significant differences due to the different treatments (**Table 3**). Rice seedlings without foliar applications of plant hormones (16.19 µmol g^-1^ (FW)) showed a higher MDA content compared to plants grown under optimal temperature conditions (4.45 µmol g^-1^ (FW)) and the rest of the treatments in the experiment. In general, rice seedlings subjected to heat stress and treated with different numbers of CK or BR sprays showed a decrease in lipid peroxidation since the MDA content was lower compared to the SC (values between 6.45 µmol g^-1^ (FW) and 7.72 µmol g^-1^ (FW)).

Proline content was lower in SC plants (1.49 µmol g^-1^ (FW)) compared to plants grown under optimal temperature conditions (AC: 2.11 µmol g^-1^ (FW)) and the rest of the treatments in the experiment. Plants with different CK applications showed a higher content of this amino acid compared to the SC treatment (values between 4.50 µmol g^-1^ (FW) and 12.50 µmol g^-1^ (FW)). BR applications increased proline contents within plants (values from 13.78 µmol g^-1^ (FW) to 9.43 µmol g^-1^ (FW)).

#### 3.2.5 Crop Stress Index (CSI) and Relative Tolerance Index (RTI)

**Table 4** shows the effect of different numbers of foliar growth regulator applications (BR vs. CK) and combined heat stress on the relative tolerance index (RTI) or crop stress index (CSI) in rice plants. The RTI showed behavior similar to the rest of the physiological variables, obtaining significant differences (P ≤ 0.01) between the different treatments (**Table 4**). SC plants showed a lower tolerance than the rest of the treatments (19.10%). Foliar sprays of plant hormones improved RTI in plants exposed to stressful temperatures. This effect was more noticeable in the ‘F2000’ plants treated with two applications of CK or three applications of BR (57.14 and 77.31%, respectively). On the other hand, differences were observed in the CSI of rice plants subjected to the stress condition and with foliar sprays of plant growth regulators (P ≤ 0.01). Rice plants only exposed to combined heat stress registered the highest index (0.93). However, the CSI was lower when the rice plants were treated with the different numbers of foliar applications of the evaluated growth regulators (values between 0.3 and 0.64). Finally, rice plants grown under optimal conditions showed a CSI of 0.56.

**Table 4:** Effect of the number of applications of two growth regulators (cytokinins (CK) or brassinosteroids (BR)) on the crop stress index (CSI) and relative tolerance index (RTI) of rice plants of genotype Federarroz 2000 subjected to combined heat stress (40°C day/30°C night) at 88 days after emergence (DAE).

Treatments	CSI	RTI (%)
Absolute control	0.56 ab	——
Heat stress control	0.93 a	19.10 d
One spray of CK	0.32 b	36.20 cd
Two sprays of CK	0.40 ab	57.14 b
Three sprays of CK	0.38 ab	44.49 bc
One spray of BR	0.49 ab	27.66 cd
Two sprays of BR	0.51 ab	32.64 cd
Three sprays of BR	0.64 ab	77.31 a
Significance	*	***

The treatments evaluated were as follows: absolute control (AC), heat stress control (SC), one foliar spray of CK (CK1), two foliar sprays of CK (CK2), three foliar sprays of CK (CK3), one foliar spray of BR (BR1), two foliar sprays of BR (BR2) and three foliar sprays of BR (BR3). Data represent the mean of five data points ± standard error (n = 5). Data followed by different letters indicate statistically significant differences according to Tukey’s test (P ≤ 0.05). Equal letters indicate that the means are not statistically significant (≤0.05). NS, *, ** or *** not significant or significant at P ≤ 0.05, 0.01 or 0.001, respectively.

### 3.3 Third experiment (E3): Evaluation of foliar applications of cytokinins or brassinosteroids on rice plants at two reproductive stages

#### 3.3.1 Photosynthetic variables: Photosynthesis (P_n_), transpiration (*E*), stomatal conductance (g_s_), intercellular carbon (C_i_), water use efficiency (WUE), carboxylation efficiency (P_n_/C_i_), F_v_/F_m_ ratio, and non-photochemical quenching (NPQ)

The values of the different photosynthetic variables measured in experiment three are summarized in **Table 5**. An interaction was observed between the phenological stage and the application treatment on the variables P_n_, WUE, P_n_/C_i_, F_v_/F_m,_ and NPQ. The treatments stress-R2 + BR3 and AC-R8 recorded the highest photosynthetic values (22.53 and 11.88 µmol CO_2_ m^-2^ s^-1^, respectively) compared to the rest of the treatments (between 4.39 and 9.38 µmol CO_2_ m^-2^ s^-1^). Regarding the WUE, the treatment stress-R2 + BR3 showed a higher relationship (90.81% vs. ± 17% for the rest of the treatments). Finally, the P_n_/C_i_ was favored by the treatments stress-R8 + CK3 (0.070) and stress-R2 + BR3 (0.036).

**Table 5:** Effect of the applications of two growth regulators (cytokinins (CK) or brassinosteroids (BR)) on the photosynthetic variables of rice plants of genotype Federarroz 2000 subjected to combined heat stress (40°C day/30°C night) at R2 (88 DAE) or R8 (108 DAE).

Treatment	P_n_	*E*	g_s_	C_i_	WUE	P_n_/C_i_	F_v_/F_m_	NPQ
**Phenological stage (S)**	***	NS	NS	NS	**	***	*	*
**Treatment (T)**	***	NS	NS	*	**	***	**	**
**Interaction (SxT)**	***	NS	NS	NS	**	***	**	**
Absolute control (AC)-R2	9.38 bc	8.19 a	0.74 a	346.78 a	37.59 b	0.026 bc	0.65 ab	0.55 d
Heat stress control (SC)-R2	9.02 bc	8.28 a	0.69 a	324.72 a	12.25 b	0.026 bc	0.61 c	1.78 a
Stress-R2 + CK3	6.71 cd	9.60 a	0.25 a	326.08 a	31.82 b	0.020 bc	0.67 a	0.98 b
Stress-R2 + BR3	22.53 a	13.20 a	0.21 a	326.29 a	90.81 a	0.036 b	0.67 a	1.12ab
Absolute control (AC)-R8	11.88 b	10.97 a	0.45 a	356.45 a	28.82 b	0.016 c	0.67 a	0.70 c
Heat stress control (SC)-R8	6.10 cd	8.70 a	0.41 a	337.15 a	14.06 b	0.010 c	0.59 c	1.84 a
Stress-R8 + CK3	4.39 d	9.11 a	0.45 a	355.83 a	17.02 b	0.070 a	0.62 b	1.21 ab
Stress-R8 + BR3	8.71 cd	10.49 a	0.49 a	337.14 a	27.53 b	0.026 bc	0.63 b	1.17 ab
**CV (%)**	15.38	30.70	86.41	4.35	44.91	19.97	3.91	4.51

Photosynthesis (P_n_), transpiration (E), stomatal conductance (g_s_), intercellular carbon (C_i_), water use efficiency (WUE), carboxylation efficiency (P_n_/C_i_), F_v_/F_m_ ratio, and non-photochemical quenching (NPQ). The treatments evaluated were as follows: absolute control (AC), heat stress control (SC), three foliar sprays of CK (CK3), and three foliar sprays of BR (BR3). Data represent the mean of five data points ± standard error (n = 5). Data followed by different letters indicate statistically significant differences according to Tukey’s test (P ≤ 0.05). Equal letters indicate that the means are not statistically significant (≤0.05). NS, *, ** or *** not significant or significant at P ≤ 0.05, 0.01 or 0.001, respectively. C.V., coefficient of variation.

For F_v_/F_m_, the SC treatment registered the lowest ratio of all the treatments (± 0.60 for both phenological stages), indicating an affectation caused by the combined heat stress. However, the plants treated with three applications of CK or BR showed an improvement in this variable, registering average values of 0.67 in R2 and 0.62 for R8 for both treatments, respectively. The NPQ showed that the SC treatments in both periods of combined heat stress obtained the highest values among the treatments (± 1.80 for both phenological stages). The treatments with three applications of CK or BR mitigated the damage in PSII since the average values recorded were 1.05 and 1.19 for R2 and R8, respectively. On the other hand, *E*, g_s,_ and C_i_ did not show significant differences between phenological stages, foliar application treatments, or their interaction.

#### 3.3.2 Characteristics of the panicle

**Table 6** also shows the differences between the phenological stage and treatments on the number of filled spikelets, blank spikelets, and percentage of panicle blanking. It was observed that a period of combined heat stress during flowering generates a greater number of blank spikelets and a higher percentage of panicle blanking. In this phenological stage, it was observed that the application of CK reduced the damage on the spikelets, resulting in a lower percentage of panicle blanking. Regarding the stage of maturation (R8), such notorious damage was not generated in the evaluated components of the panicles and the average values of the number of blank spikelets and the percentage of panicle blanking were lower than the treatments in the reproductive phase.

**Table 6:** Effect of the applications of two growth regulators (cytokinins (CK) or Brassinosteroids (BR)) on photosynthetic variables of rice plants of the genotype Federarroz 2000 subjected to combined heat stress (40°C day/30°C night) at 88 and 120 days after emergence (DAE).

	Filled spikelets	Blank spikelets	% of panicle blanking
**Phenological stage (S)**	***	***	***
**Treatment (T)**	*	*	**
**Interaction (SxT)**	*	**	**
Absolute control (AC)-R2	89.25 b	18.00 b	17.94 c
Heat stress control (SC)-R2	48.33 b	68.33 a	59.85 a
Stress-R2 + CK3	85.50 b	30.00 ab	25.67 bc
Stress-R2 + BR3	138.00 a	52.75 ab	49.69 ab
Absolute control (AC)-R8	146.40 a	10.40 b	6.62 c
Heat stress control (SC)-R8	121.00 b	11.00 b	8.18 c
Stress-R8 + CK3	100.00 c	14.75 a	11.59 a
Stress-R8 + BR3	138.00 a	13.75 a	10.94 b
**CV (%)**	24.38	54.32	43.19

The treatments evaluated were as follows: absolute control (AC), heat stress control (SC), three foliar sprays of CK (CK3), and three foliar sprays of BR (BR3). Data represent the mean of five data points ± standard error (n = 5). Data followed by different letters indicate statistically significant differences according to Tukey’s test (P ≤ 0.05). Equal letters indicate that the means are not statistically significant (≤0.05). NS, *, ** or *** not significant or significant at P ≤ 0.05, 0.01 or 0.001, respectively. C.V., coefficient of variation.

## 4 Discussion

The results of the different experiments in this study showed that the different applications of CK or BR helped the acclimatization of rice plants to the exposure to combined heat stress. The degree of acclimatization was assessed using physiological and biochemical variables (photosynthesis (P_n_), stomatal conductance (g_s)_, leaf temperature, F_v_/F_m_ ratio, photosynthetic pigments, percentage of panicle blanking, water use efficiency (WUE), and proline and nutrient contents).

Foliar sprays of the two growth regulators (1, 2, or 3 foliar applications) increased gas exchange properties of leaves (g_s_ or P_n_) and reduced leaf temperature under combined heat stress according to the experiment. Previous studies also showed that the foliar application of biostimulants, nutrients, or hormones had a positive effect on these variables in plants exposed to heat stress due to high daytime temperatures, concluding that g_s_, P_n,_ or leaf temperature are reliable physiological variables to evaluate the use of agronomic strategies to mitigate the effects of high-temperature stress (Calderon-Paez et al., 2021; Quintero-Calderon et al., 2021) An increase in gas exchange properties and a decrease in temperatures are due to the fact that plant growth regulators (CK or BR) are involved in the interaction with the synthesis of secondary osmolytes (proline) and other signaling hormones such as ABA, which is linked to stomatal closure under stress conditions (Mackova et al., 2013; Zhou et al., 2014). These growth regulators (CK or BR) have also been reported to help protect the photosynthetic apparatus through increased synthesis of antioxidant enzymes (superoxide dismutase, catalase, and ascorbate peroxidase), which are responsible for degrading reactive oxygen species (ROS). The use of these compounds (CK or BR) promotes a greater leaf gas exchange since it favors greater stomatal opening, improving the water flow inside the plant and regulating leaf temperature (Sonjaroon et al., 2018; Quintero-Calderón et al., 2021).

The use of the F_v_/F_m_ ratio is a simple technique that allows estimating plant responses to both abiotic and biotic stress conditions (Chaerle et al., 2007; Kalaji et al., 2017). In the three experiments carried out, plants of the SC treatment showed the lowest values of the F_v_/F_m_ ratio. Najeeb et al. (2021) and Quintero-Calderon et al. (2021) also found that the F_v_/F_m_ ratio decreased significantly in rice leaves under heat stress. A drop in the F_v_/F_m_ ratio during high-temperature stress suggests a decrease in energy capture and conversion in the PSII, indicating disorganization of the PSII (Feng et al., 2013). In general, the use of BR or CK markedly improved PSII activity when at least two applications were made to stressed plants in the different experiments. It has also been observed that two applications of BR improved PSII activity under heat stress in rice plants (Thussagunpanit et al., 2015). This may be associated with the fact that plants sprayed with BR generate a greater amount of heat shock proteins, which accumulate and protect PSII (Kothari and Lachowiec, 2021). Regarding the use of CK, Kumar et al. (2020) also found that foliar applications of CK enhance PSII activity through increased zeaxanthin activity.

Non-photochemical quenching (NPQ) is another parameter of chlorophyll α fluorescence that is an indicator of energy dissipation as heat under ambient conditions (Chaudhary et al., 2020). The plants that were only subjected to combined heat stress without the application of any of the hormones in the experiments showed the highest values of NPQ. Qu et al. (2021) obtained similar results when rice plants exposed to heat stress of 40°C showed higher NPQ compared to plants under optimal conditions (25°C). Rice plants subjected to combined heat stress and treated with exogenous applications of CK or BR showed a decrease in NPQ (**Figures 2B** and **5B**), indicating that these hormones helped plant acclimatization by dissipating energy in the form of heat. It has been observed that foliar applications of CK or BR also caused a reduction in NPQ under conditions of abiotic stress (water or saline) in lulo and melon, suggesting that these regulators play an important role in energy dissipation and photosynthesis optimization (Wu et al., 2012; Castañeda-Murillo et al., 2022). Better performance of the F_v_/F_m_ and NPQ parameters in plants treated with CK or BR under combined heat stress may be due to the fact that these compounds can favor the synthesis of heat shock proteins or antioxidant enzymes. Additionally, CK and BR reduce the loss of photons, mainly in the form of heat, optimizing the use of light in photochemical processes (Kothari and Lachowiec, 2021; Castañeda-Murillo et al., 2022).

Most of the abiotic stresses can generate changes in the contents of photosynthetic pigments in leaves (Chen et al., 2017). In general, rice plants exposed only to combined heat stress showed a lower content of photosynthetic pigments in the first two experiments. A decrease in chlorophyll content was also observed when wheat plants were subjected to high-temperature stress conditions (greater than 35°C) for three days (Feng et al., 2013). Fahad et al. (2017) explain that heat stress can inhibit biosynthesis, accelerate degradation, or generate a combined effect on chlorophyll production, causing a lower content of this photosynthetic pigment in leaves. Two or three foliar applications of CK or BR improved chlorophyll content in plants of the genotype F2000 under combined heat stress. Similar results were observed when rice plants were also treated with two exogenous applications of zeatin or epibrassinolide under heat stress conditions (Veerasamy et al., 2007; Thussagunpanit et al., 2015). Liu et al. (2020) mention that CKs protect the chlorophyll content because they inhibit leaf senescence through the expression of heat shock proteins (HSP18) under heat stress. Sharma et al. (2017) also report that BR can protect leaf chlorophylls by inducing the synthesis of different enzymes involved in chlorophyll biosynthesis under heat stress. BR also favor the expression of heat shock proteins that protect existing chlorophylls and help metabolic acclimatization (Sharma et al., 2017; Liu et al., 2020).

Malondialdehyde and proline levels are useful to understand acclimatization processes or the effect of applications or agronomic practices when plants are exposed to stress (Ahmed and Hassan, 2011; Tang et al., 2018). In general, CK or BR applications helped to decrease MDA content and increase proline synthesis, mainly when plants were treated with two or three foliar sprays. Zhang and Ervin (2008) and Quintero-Calderon et al. (2021) observed that MDA production decreased in creeping bentgrass and rice plants exposed to heat stress conditions and treated with a cytokinin-based seaweed extract or a brassinosteroid analog, respectively. Both hormones can promote the synthesis of osmoprotectants, such as betaine, proline, or ectoine that help mitigate the effects of different stresses (Liu et al., 2020; Kothari and Lachowiec, 2021).

Heat stress often decreases the concentration of nutrients in plant tissues or the total nutrient content in plants (Hussain et al., 2019). In this study, the SC treatments were those that obtained the lowest concentration of nutrients (N, K, and Ca) of all the treatments. A reduction in the content of N, P, K, and Ca was also observed in rice (Chaturvedi et al., 2017) and quinoa (Rodríguez et al., 2021) plants under stress caused by high temperatures. A reduction in the nutrient content under heat stress conditions may be because this type of stress can affect the enzymes involved in nitrogen assimilation (nitrate reductase activity) (Huang et al., 2012; Sehgal et al., 2018), and cause the loss of inorganic nutrients due to damage to the integrity of membranes (Wahid et al., 2007) or under mass flow (Fahad et al., 2017; Giri et al., 2017). Two or three sprays of BR or CK favored the concentration of nutrients (N, K, and Ca) in plants exposed to combined heat stress. Wang et al. (2013) also observed that the use of CK favored the nutritional status of nitrogen during heat stress in creeping bentgrass. Praveena et al. (2020) report that the use of brassinosteroids favors nutrient content in plants under multiple abiotic stresses. This result may be due to the fact that these compounds increase the contents of osmolytes within the roots, favor mass flow, prevent the degradation of proteins responsible for nitrogen uptake, and promote the expression of transport channels (Liu et al., 2020; Praveena et al., 2020; Kothari and Lachowiec, 2021).

The characteristics of the panicle are a great tool to determine plant behavior under heat stress (Abdelaal et al., 2021). The plants that were exposed to combined heat stress in R2 showed a higher percentage of panicle blanking than the plants at stage R8. Wang et al. (2019) also observed a higher percentage of panicle blanking when plants were exposed to heat stress during flowering (≥ 35°C). Foliar sprays of CK or BR favored the number of filled spikelets and decreased the percentage of panicle blanking mainly in rice plants subjected to heat stress in the R2 stage. Similar results were also observed by Janeczko et al. (2010) and Yang et al. (2016) who found that the exogenous application of BR or CK favored yield components of the spikelet of barley or wheat, respectively. It is inferred that these hormones helped mitigate damage to the reproductive organs since they are involved in panicle differentiation, pollen tube elongation, and flower production by regulating processes such as cell division and biosynthesis of proteins and nucleic acids (Waisi et al., 2018; Yang et al., 2021).

The crop stress index (CSI) and relative tolerance index (RTI) are used to determine the effectiveness of stress (abiotic and biotic) mitigation strategies (Castro-Duque et al., 2020; Chavez-Arias et al., 2020). Foliar sprays of BR or CK caused a decrease in CSI under abiotic stress (0.3-0.5) compared to SC rice plants (0.8-0.9). In a previous experiment, similar behavior was observed for this index (Pantoja-Benavides et al., 2021). Lee et al. (2010) observed that two cotton cultivars under water stress showed a CSI of 0.85, whereas well-irrigated cultivars recorded CSI values between 0.4 and 0.6. This suggests that CSI may be an indicator of cultivar adaptability to water stress conditions. The RTI also showed a greater increase with the use of two and three applications of CK or BR compared to SC, indicating that these compounds helped improve the tolerance of rice to combined heat stress. Chavez-Arias et al. (2020) evaluated the effect of synthetic elicitors as a strategy to mitigate combined stress (biotic and abiotic) in cape gooseberry plants, finding that plants sprayed with these compounds showed a higher RTI compared to plants only exposed to the stressful condition.

Studies on rice in Colombia have initially focused on the evaluation of genotypes with resistance to high daytime or nighttime temperatures using physiological or biochemical traits (Sánchez-Reinoso et al., 2014; Alvarado-Sanabria et al., 2021; Quintero-Calderon et al., 2021). In recent years, practical, economic, and profitable techniques have been sought to propose integrated crop management and, thus, mitigate the effects of periods of heat stress in the country (Calderon-Paez et al., 2021; Quintero-Calderón et al., 2021). For this reason, the physiological, biochemical, and characteristic responses of the panicle of rice plants to combined heat stress (40°C day/30°C night) observed in this experiment indicate that the use of two or three sprays of CK or BR may be an additional technique to consider when attempting to mitigate the adverse effects of moderate periods of heat stress in rice crops.

In conclusion, Fedearroz 2000 rice plants are susceptible to a moderate period of combined heat stress because plants without foliar applications had a higher oxidative stress damage (high MDA content). The results obtained suggest that foliar cytokinins or brassinosteroids applications (between two and three applications) enhanced plant tolerance since these treatments increased the leaf chlorophyll content, leaf exchange properties (g_s_ and P_n_), fluorescence parameters of chlorophyll α (F_v_/F_m_ ratio), and decreased the MDA content, NPQ, and canopy temperature. This allows us to conclude that the use of foliar cytokinins or brassinosteroids applications can be considered as an agronomic tool to prepare rice plants when periods of high temperatures are expected during their crop development.

## Data availability statement

The raw data supporting the conclusions of this article will be made available by the authors, without undue reservation.

## Author contributions

AP-B and HR-D: conceptualization and writing – review and editing. AP-B, HR-D, and GG-V: methodology, investigation, and writing – original draft. AP-B, HR-D, and GG-V: validation. AP-B and HR-D: formal analysis. AP-B and HR-D: data curation. HR-D and GG-V: resources, supervision, project administration, and funding acquisition. All authors agreed to be accountable for the content of the work.

## Funding

This research was partially supported by the “Convocatoria para la Financiación Parcial de Proyectos de Tesis de Doctorado y Maestría” of the Facultad de Ciencias Agrarias, Universidad Nacional de Colombia, Campus Bogotá.

## Conflict of interest

The authors declare that the research was conducted in the absence of any commercial or financial relationships that could be construed as a potential conflict of interest.

## Publisher’s note

All claims expressed in this article are solely those of the authors and do not necessarily represent those of their affiliated organizations, or those of the publisher, the editors and the reviewers. Any product that may be evaluated in this article, or claim that may be made by its manufacturer, is not guaranteed or endorsed by the publisher.
